# DEAR4, a Member of DREB/CBF Family, Positively Regulates Leaf Senescence and Response to Multiple Stressors in *Arabidopsis thaliana*

**DOI:** 10.3389/fpls.2020.00367

**Published:** 2020-03-31

**Authors:** Zenglin Zhang, Wei Li, Xiaoming Gao, Mengmeng Xu, Yongfeng Guo

**Affiliations:** Tobacco Research Institute, Chinese Academy of Agricultural Sciences, Qingdao, China

**Keywords:** *DEAR4*, leaf senescence, stress, ROS, COR, RD, *Arabidopsis thaliana*

## Abstract

Leaf senescence is a programmed developmental process regulated by various endogenous and exogenous factors. Here we report the characterization of the senescence-regulating role of *DEAR4* (AT4G36900) from the DREB1/CBF (dehydration-responsive element binding protein 1/C-repeat binding factor) family in Arabidopsis. The expression of *DEAR4* is associated with leaf senescence and can be induced by ABA, JA, darkness, drought and salt stress. Transgenic plants over-expressing *DEAR4* showed a dramatically enhanced leaf senescence phenotype under normal and dark conditions while the *dear4* knock-down mutant displayed delayed senescence. *DEAR4* over-expressing plants showed decreased seed germination rate under ABA and salt stress conditions as well as decreased drought tolerance, indicating that DEAR4 was involved in both senescence and stress response processes. Furthermore, we found that DEAR4 protein displayed transcriptional repressor activities in yeast cells. DEAR4 could directly repress the expression of a subset of *COLD-REGULATED* (*COR*) and *RESPONSIVE TO DEHYDRATION* (*RD*) genes which have been shown to be involved in leaf longevity and stress response. Also we found that DERA4 could induce the production of Reactive oxygen species (ROS), the common signal of senescence and stress responses, which gives us the clue that DEAR4 may play an integrative role in senescence and stress response via regulating ROS production.

## Introduction

Senescence is the last stage of leaf development which is influenced by intrinsic and environmental factors including age, nutrients, hormones, darkness, osmotic stress, extreme temperature and pathogens (Lim et al., 2007; Guo and Gan, 2014). Most of the major plant hormones have been reported to affect leaf senescence process: abscisic acid (ABA), ethylene (ETH), jasmonic acid (JA), salicylic acid (SA) and strigolactones function in promoting senescence, while cytokinins (CK), gibberellic acid (GA) and auxin inhibit senescence (Jibran et al., 2013; Li et al., 2013; Penfold and Buchan-Wollaston, 2014; Mostofa et al., 2018). During the process of leaf senescence, cellular metabolism and structure undergo significant changes, resulting in leaf yellowing. Meanwhile, degradation of macromolecules in senescing leaves functions to remobilize nutrients to support young vegetative organs and reproductive growth. As a mechanism of evolutional fitness, unfavorable environmental conditions can induce precocious senescence leading to reduced yield and quality of crop plants (Wu et al., 2012; Gregersen et al., 2013; Zhang and Zhou, 2013; Yolcu et al., 2017). Senescence execution requires differential expression of a large number of genes a subset of which is called senescence-associated genes (*SAGs*). A number of *SAGs* have been identified to play a regulatory role in leaf senescence. These include genes encoding transcription factors of WRKY, NAC, DREB, MYB, and bZIP family (Woo et al., 2001; Yang et al., 2011; Lee et al., 2012; Vainonen et al., 2012).

Dark-induced senescence (DIS) has been widely used as a model system in leaf senescence study (Liebsch and Keech, 2016). Differential expression of a large number of transcription factor genes during dark-induced leaf senescence have been reported (Song, 2014; Song et al., 2014; Yasuhito et al., 2014) and some of them have been studied for their function in regulating senescence. *AtWRKY22* is induced by darkness, but suppressed by light. Further study reveals that *AtWRKY22* over-expressing plants displayed accelerated senescence, whereas *AtWRKY22* loss-of-function plants showed a delay of senescence under dark condition (Zhou et al., 2011b). *RD26* loss-of-function plants displayed significantly delayed senescence under normal or dark-induced conditions with a higher chlorophyll level detected. By contrast, over-expression of *RD26* led to early leaf senescence (Takasaki et al., 2015; Kamranfar et al., 2018). Over-expression of *CBF2* and *CBF3*, two members of the DREB family in Arabidopsis, significantly delayed the onset of leaf senescence and also delayed leaf senescence induced by hormones and darkness (Sharabi-Schwager et al., 2010a, b). Phytochromes regulate light responses by promoting the degradation of PIFs (Phytochrome-interacting factors). PIF3, 4, and 5 play an important role in natural and dark induced senescence (Yasuhito et al., 2014). Mutations of the *PIFs* genes resulted in a significant delay of natural and dark induced senescence, whereas over-expression of these genes accelerated senescence. Further study revealed that PIF4 can bind the promoter of *NYE1*, the chlorophyll degradation regulatory gene, and *GLK2*, the chloroplast activity maintainer gene, resulting in induction and repression of their expression, respectively (Song et al., 2014; Shi et al., 2017a).

Jasmonic acid is involved in multiple processes including root inhibition, trichome initiation, anthocyanin accumulation, leaf senescence and biotic and abiotic stress responses (Balbi and Devoto, 2008; Wu et al., 2008; Hu et al., 2017; Song et al., 2017; Ono et al., 2019). JA signaling can be initiated by perception of jasmonoyl-L-isoleucine (JA-Ile), which binds to its receptor COI1 (CORONATINE INSENSITIVE1), an F-box domain-containing protein (Balbi and Devoto, 2008; Shan et al., 2011). It has been reported that endogenous JA content increases during leaf senescence and JA biosynthetic genes such as *LOX1*, *LOX3*, and *LOX4* are up-regulated during leaf senescence (Wasternack, 2007; Seltmann and Berger, 2013). Attached or detached leaves displayed precocious senescence under exogenous application of MeJA (Shan et al., 2011; Hu et al., 2017). Further study revealed that MYC2, 3, and 4 redundantly bind to the *SAG29* promoter and activate its expression, leading to activation of JA-induced leaf senescence (Zhu et al., 2015). In contrast, the bHLH family members including bHLH03, bHLH13, bHLH14, and bHLH17 attenuate MYC2/MYC3/MYC4-activated JA-induced leaf senescence by binding to the promoter of *SAG29* and repress its expression. It has been suggested that the activators and repressors mediated in JA-induced leaf senescence can enhance the plant survival rate in various environmental conditions (Qi et al., 2015). In addition, the NAC transcriptional factors *ANAC019*, *ANAC055*, and *RD26* are also direct targets of MYC2 in mediating JA-induced leaf senescence (Zhu et al., 2015). The JA signaling proteins JAZ4 and JAZ8 interact with transcription factor WRKY57 to negatively regulate leaf senescence induced by JA (Jiang et al., 2014). Additionally, the expression of *JAZ7* was significantly increased during darkness. *jaz7* mutant exhibited precocious senescence induced by darkness, suggesting that *JAZ7* plays a negative role in dark-induced leaf senescence (Yu et al., 2016). The Evening Complex (EC), a core component of the circadian oscillator, comprising EARLY FLOWERING3 (ELF3), EARLY FLOWERING4 (ELF4) and LUX ARRHYTHMO (LUX), plays essential roles in the plant circadian clock and negatively regulates leaf senescence in Arabidopsis. It has been reported that EC represses the expression of *MYC2* by directly binding to its promoter. *myc2myc3myc4* triple mutants abrogate the accelerated leaf senescence induced by JA in EC mutants (Zhang et al., 2018; Thines et al., 2019). Additionally, JA can positively regulate the ICE-CBF signal to enhance cold stress tolerance in Arabidopsis. Interestingly, endogenous JA content was increased under cold stress conditions. Exogenous application of MeJA enhanced cold stress tolerance. Further study revealed that JAZ1 and JAZ4 play negative roles in the ICE-CBF pathway (Hu et al., 2013, 2017).

As sessile organisms, plants have developed sophisticated mechanisms that are activated and integrated by the expression of thousands of genes to cope with variety of environmental stresses (Yeung et al., 2018; Asad et al., 2019). Many transcriptional factors were found to play key roles in stress response and tolerance. As the largest transcription factor family in Arabidopsis, the AP2/ERF family contains 147 members functionally categorized into the development-associated AP2 and RAV subgroups and the stress response-associated DREB and ERF subgroups (Liu et al., 1998; Sakuma et al., 2002). The DREB/CBF proteins are known to directly regulate target genes in response to various stresses including high salinity, drought and cold stress by directly binding the conserved DRE (Dehydration responsive element)/CRT(C-repeat) *cis-*acting regulatory elements which contains the core sequence CCGAC (Sakuma et al., 2002; Sun et al., 2008). DREB homologs have been identified in a variety of plants species including rice (Dubouzet et al., 2003), cotton (Huang and Liu, 2006; Ma et al., 2014) and soybean (Mizoi et al., 2013). In Arabidopsis, there was 57 DREB transcription factors classified into six groups termed A-1 to A-6 based on the similarities of the AP2/ERF domain (Sazegari et al., 2015). DREB1 including CBF1, CBF2, and CBF3 belongs to the A-1 clade that play an important role in cold stress response and dark induced senescence process (Xu et al., 2010; Wang et al., 2018). Over-expression of *CBF1* or *CBF3* can also confer plant more capacity of freezing tolerance (Novillo et al., 2004; Zhou et al., 2011a). DREB2 proteins belong to the A-2 clade which is involved in regulation of drought and heat response. Over-expression of *DREB2A* improved survival rate under drought or heat stress (Schramm et al., 2008). In the A-6 clade, *RAP2.4* was induced by salt stress. Over-expression of *RAP2.4* can enhance drought tolerance in Arabidopsis. Moreover, RAP2.4A is involved in regulating expression of several chloroplast-targeted antioxidant genes. The expression of *RAP2.4B* was increased under heat stress conditions. Plants over-expressing *RAP2.4B* or *RAP2.4* were hypersensitive to exogenous ABA at germination. Over-expression of *Rap2.4f* (*At4g28140*) caused precocious senescence according to the detection of increasing chlorophyll degradation and up-regulation of many *SAGs* (Lin et al., 2008; Xu et al., 2010; Rae et al., 2011).

There are six *DEAR* genes named *DEAR1* to *DEAR6* within the Arabidopsis genome that contain sequences with homology to the DREB domain and EAR motif. Plants over-expressing *DEAR1* displayed phenotypes of cell death, increased resistance to pathogen infection and reduced freezing tolerance. Additionally, DEAR1 suppressed the expression of *DREB1/CBF* family genes induced by cold treatment, which resulted in reduced freezing tolerance (Wang et al., 2008; Tsutsui et al., 2009). *DEAR4* was identified as a regulator of cell death in the hypocotyls-root transition zone using the inducible over-expression strategy (Coego et al., 2014). In this study, we characterize the function of DEAR4, a member of DREB/CBF family, in leaf senescence and stress response. The expression of *DEAR4* was strongly induced by developmental stage, darkness and multiple stresses. Phenotype analysis revealed that DEAR4 was involved in the senescence process induced by age and darkness. Further study revealed that over-expression of *DEAR4* led to reduce expression of *COR* and *RD* genes which are involved in senescence and stress regulation. Our findings suggest that DEAR4 is a transcriptional repressor and plays a role in regulating leaf senescence and stress response by repressing the expression of *COR* and *RD* genes.

## Materials and Methods

### Plant Materials, Growth Conditions

Arabidopsis seeds were surface-sterilized by 75% (v/v) ethanol followed by 3 washes with water. Then the seeds were sown on 0.5× Murashige and Skoog medium (MS) and kept at 4°C for 3 day. One-week-old seedlings were transferred into soil. Plants were grown in growth chambers at 22°C under continuously light. The mutant *dear4* (*Salk_010653c*), *dear4-1*(*Salk_045347*) and *DEAR4* inducible over-expression lines *DEAR4-ind-1*(*cs2102284*) and *DEAR4-ind-2*(*cs2102286*) used in this study were obtained from the Arabidopsis Biological Resource Center (ABRC).

### Detached Leaf Phenotype Investigation

For natural leaf senescence evaluation, the fifth and sixth rosette leaves of 4-week-old plants were detached for measurements of chlorophyll content and ion leakage rate. In the hormone induced leaf senescence assay, the fifth and sixth rosette leaves of 4-week-old plants were detached and floated on 3 mL of treatment buffer (0.5× MS, 3 mM MES, PH: 5.8) supplemented with 50 μM MeJA. Petri dishes were sealed with parafilm tape, wrapped with double-layer aluminum foil. In the dark induced senescence assay, the fully expanded fifth and sixth rosette leaves were detached from 4-week-old plants grown in soil. Then, detached leaves were placed in petri dishes containing two layers of filter paper soaked in 10 mL of treatment buffer (0.5× MS, 3 mM MES adjust PH to 5.8). Then the petri dishes were wrapped in aluminum foil for 5 days. Three biological replicates were performed.

### Chlorophyll Content, Ion Leakage Measurement

For chlorophyll content measurement, detached leaf was weighted and soaked in 96% (v/v) ethanol (3–4 mg of tissue in 1 mL of ethanol) overnight at room temperature in the dark condition. Total chlorophyll content was determined by measuring the absorbance at 646.6 and 663.6 nm as described (Zhang and Guo, 2018). For ion leakage measurement, detached leaves were washed three times with deionized water followed by immersion in deionized water, then gentle shaking for 1–2 h at room temperature. Total conductivity was measured as initial readings data, then samples were boiled and cooled down to room temperature and measured again with a bench-top conductivity meter (CON500, CLEAN Instruments) to get the final total conductivity. Total electrolyte leakage is determined by the following formula: Ions leakage (%) = initial / final conductivity ^∗^100 (Zhang and Guo, 2018). Three biological replicates were performed.

### Hormone and Stress Treatments for Gene Expression Analysis

The fifth leaf was detached from 4-week-old plants grown in continuously light condition, then transferred into 0.5× MS liquid culture containing the plant hormones ABA (1 μM), SA (20 μM), IAA (20 μM), MeJA (20 μM), ACC (20 μM) or GR24 (5 μM), incubated for 6 and 8 h, respectively. In environmental condition treatments, the leaves were transferred into 0.5× MS liquid culture containing NaCl (100 mM), mannitol (200 mM) or transferred to 4°C from room temperature for 12 and 24 h.

### qRT-PCR

Total RNA was isolated from frozen leaf samples using trizol according to instructions of the manufacturer. The RNA was treated with RNase-free DNaseI to release the genomic DNA. First-strand cDNAs were generated from total RNA by reverse transcription using an AMV reverse transcriptase first-strand cDNA synthesis kit (Life Sciences, Promega). Next, cDNA samples were used for the following qRT-PCR. Triplicate quantitative assays and three biological replicates were performed using the SYBR Green Master mix using an ABI 7500 sequence detection system (Applied Biosystems). The relative quantitation method (^ΔΔ^CT) was used to evaluate quantitative variation among replicates. *Actin2* were applied as internal controls to normalize all data. The primers used in this study are as follows: Q_*SAG12*_F:5′-TCCAATTCTATTCGTCTGGTGTGT-3′; Q_*SAG12*_R: 5′-CCACTTTCTCCCCATTTTGTTC-3′; Q_*SEN4*_F: 5′-GACTC TTCTCGTGGCGGCGT-3′;Q_*SEN4*_R 5′-CCCACGGCCATTC CCCAAGC-3′ Q_*ACT2*_F: 5′-TGTGCCAATCTACGAGGGTT T-3′; Q_*ACT2*_R: 5′-TTTCCCGCTCTGCTGTTGT-3′; Q_*RBC S3B*_F: 5′-AGTAATGGCTTCCTCTATGC-3′; Q_*RBCS3B*_R: 5′-GTGATGTCCTTGTTGGTCTTG-3′; Q_*DEAR4*_F:5′-GAGG TCCTTCTGCTCGGCTT-3′; Q_*DEAR4*_R:5′-CCGCCGACAT ATCTCCACCA-3′.

### Stress Response Assays

For seed germination assays in different stress conditions, seeds of *DEAR4* over-expression and Col-0 were surface-sterilized and incubated in 70% (v/v) ethanol for 5 min, and then washed five times quickly with water. Then, seeds were distributed on 0.5× MS solid media supplemented with 0 mM, 50 mM, 80 mM NaCl or 0.5 μM, 0.7 μM ABA. Seeds were stratified at 4°C for 3 days then transferred to 22°C under continuously light condition. Germination was monitored every 24 h as percentage of seeds with radicles completely penetrating the seed coat, for up to 5 days. Representative graphs are shown indicating germination up to 5 days. For drought adaption measurement assay, seeds of *DEAR4* over-expression and Col-0 were sowed into soil and normally watered for 4 weeks. Then, phenotypes were observed after an additional 10 days without watering. When the Col-0 plants showed lethal phenotypes, watering was resumed, and phenotypes were observed again after an additional 5 days. The survival rate was measured based on three replicates.

### Inducible Expression of *DEAR4*

A total of 3-week-old plants grown in pots were sprayed with 10 μM β-estradiol (EST) once a day for 2 days and incubated for 6 additional days. Phenotype analysis and chlorophyll content measurement were carried out as above described. Three biological replicates were performed.

### Determination of H_2_O_2_ Accumulation

For NBT staining, the 5th leaves of 4-week old plants were detached, then, soaked in the NBT staining buffer (0.5 mg/mL NBT in 10 mM potassium phosphate buffer, pH 7.6) overnight. Leaf chlorophyll was removed in the fixative solution (ethanol: acetic acid: glycerol, 3:1:1) and then kept in the ethanol: glycerol (4:1) solution at 4°C until photographed. Endogenous hydrogen peroxide content was measured following the instructions of the manufacturer (Nanjing Jiangcheng Company, China). Three biological replicates were performed.

### Dual-Luciferase (LUC) Assay

In the dual-LUC reporter assay, *CaMV35S*:*DEAR4* was used as the effector construct. The reporter construct pGreenII 0800-LUC harboring the firefly LUC driven by promoters of *COR15a*, *COR15b*, *RD29a* or *RD29b* which contains the *DRE/CRT* element with the length of 481 bp, 361bp, 415 bp, and 429 bp, respectively. *Renilla LUC* gene driven by the *CaMV35S* promoter was used as an internal control. The reporter construct was co-transformed with the helper plasmid p19 into Agrobacterium GV3101. The Agrobacterium culture harboring the reporter construct was either incubated alone or as a mixture with the Agrobacterium culture containing the effector construct, and infiltrated manually into the leaves of *Nicotiana benthamiana*, then the plants were moved into darkness for 3 days. Firefly and Renilla luciferase activities were measured using the Dual Luciferase Reporter Assay System (Promega) according to the instruction of the manufacturer. The Firefly/Renilla luciferase ratio indicates transcriptional activity. Three biological replicates were performed.

### *proDEAR4*::*GUS* Construct and GUS Activity Detection

Firstly, we cloned the 1.4 kb promoter of *DEAR4* using primers: pro*DEAR4*-EcoRF:5′-CCGGAATTCattaccgcctcttccct att-3′ and pro*DEAR4*-NcoIR:5′-CATGCCATGGagtggttttc tccggagatttc-3′, then the empty construct pcambia3301 was digested by restriction enzymes Hind III and Nco I, forming the *proDEAR4*::*GUS* construct after ligation reaction using T4 ligase (NEB No. M0202). According to the method described by Jefferson et al. (1987), leaves at different developmental stages were detached, then immersed in the histochemical staining buffer (1 mM 5-bromo-4-chloro-3-indolyl-b-glucuronic acid (Gluc) solution in 100 mM sodium phosphate, pH 7.0, 10 mM EDTA, 0.5 mM potassium ferricyanide, 0.5 mM potassium ferricyanide and 0.1%Triton X-100), and then incubated at 37°C for 12 h. The leaves were destained in 70% ethanol before photographed.

### Transcriptional Repression Assay in Yeast Cells

To detect the transcriptional repression activity of DEAR4, yeast one-hybrid assay was employed in this study. The DEAR4-BD, shDEAR4 (without EAR domain)-BD, DEAR4-BD-VP16, shDEAR4-BD-VP16 constructs were transformed into the yeast strain Y190. To measure the strength of the X-Gal activity, liquid assay was carried out using CPRG as substrate. Three biological replicates were performed.

### Plasmid Construction and Transformation of Arabidopsis

To generate the *DEAR4* over-expression constructs, the full length of *DEAR4* CDS was amplified by nest PCR method using primers (First round primers: DEAR4-BP-F: 5′-TACAAAAAAGCAGGCTTCATGGAGACGGCGACTGAAGT GG-3′; DEAR4-BP-R:5′-GTACAAGAAAGCTGGGTCATCGT CATCTGAAGTTTCCGG-3′; second round primers: attB-F:5′-GTGGGGACAAGTTTGTACAAAAAAGCAGGCTTC-3′; attB-R:5′-GTGGGGACCACTTTGTACAAGAAAGCTGGGTC-3′). According to the instructions of the invitrogen gateway kit (kit No.11789 (BP Clonase); No.117910 (LR Clonase), the PCR products were cloned into pDnor-207 vector using the BP enzyme. Subsequently, according to Earley et al. (2006), *DEAR4* CDS was sub-cloned into pEarleyGate202 using the LR enzyme to form the *35S*::*DEAR4* construct. Then the constructs was transformed into *Agrobacterium tumefaciens* strain GV3101. The binary constructs were transformed into Arabidopsis plants via the floral dip method (Clough and Bent, 1998). Transgenic plants were selected by glyphosate resistance.

## Results

### The Expression Pattern of *DEAR4*

**FIGURE 1:**
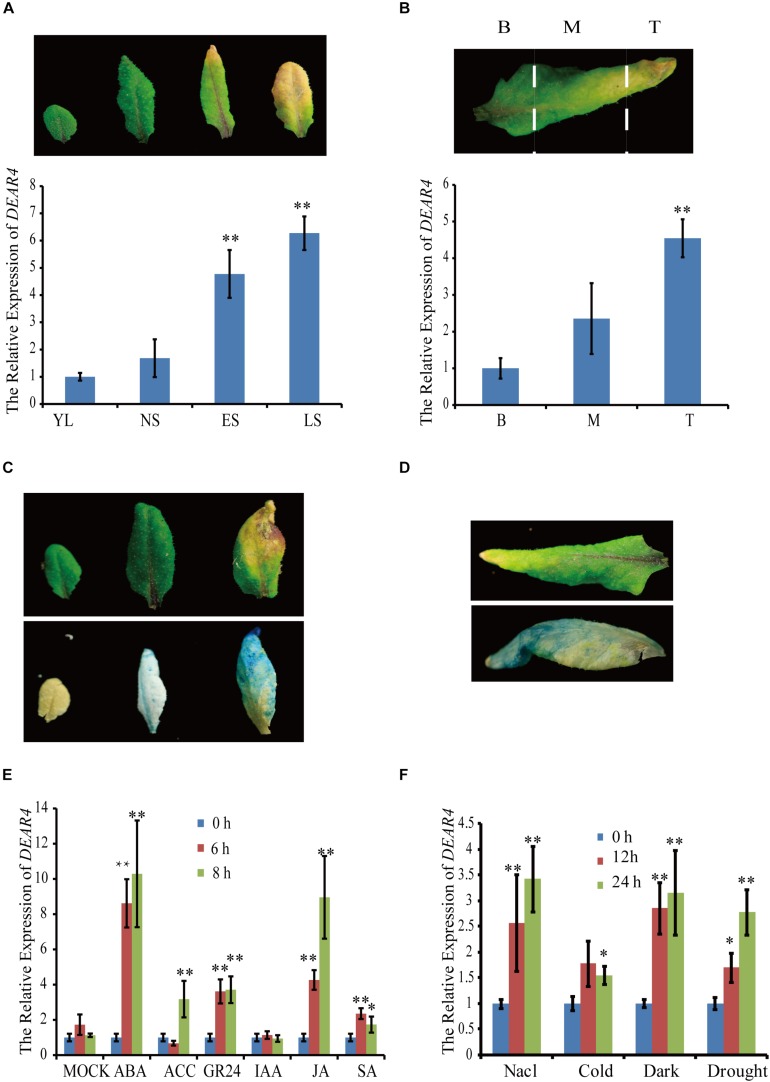
The expression pattern of *DEAR4*. **(A)** The expression pattern of *DEAR4* at different developmental stages. YL, young leaves of 2-week old seedlings; NS, fully expanded mature leaves without senescence symptoms; ES, early senescent leaves, with <25% leaf area yellowing; LS, late senescent leaves, with >60% leaf area yellowing. **(B)** The *DEAR4* expression pattern in different parts of a senescing leaf; B, Base; M, Middle; T, Tip. **(C)** *GUS* expression detection in different stage rosette leaf of *proDEAR4*::*GUS* transgenic plants. Top Leaf, before GUS staining; bottom leaf, after GUS staining. **(D)** GUS staining results in different parts of a senescing leaf. **(E)** Effects of plant hormones on *DEAR4* expression. **(F)** Effects of different stress conditions on *DEAR4* expression. The bars are standard deviations (SD) of three biological replicates. The one and double asterisks indicate significant difference to control at the levels of 0.01 < *P* < 0.05 and *P* < 0.01 using student’s *t*-test, respectively.

To identify new genes regulated by both senescence and light, the GENEVESTIGATOR database^1^ was screened and *DEAR4* was found to be highly expressed in senescing plant tissues, meanwhile induced by dark conditions. To confirm the *in silico* data, we performed qRT-PCR to measure the expression of *DEAR4*. The results showed that expression levels of *DEAR4* increased significantly from young leaf to late senescence stage (Figure 1A). In Arabidopsis, senescence proceeds from the tip toward the base of a leaf. When approximately 30% of the leaf area was yellow, the sixth leaves from 4-week-old Arabidopsis plants were detached and dissected into three parts including basal, middle and tip parts (Figure 1B). The expression of *DEAR4* in these three parts was determined by qRT-PCR and the results showed that expression of *DEAR4* exhibited higher in the tip but lower in the base region of these senescing leaves (Figure 1B).

To further examine the expression pattern of *DEAR4*, we generated *proDEAR4*::*GUS* transgenic plants that harbor the 1.4 kb long *DEAR4* promoter driving the *GUS* coding sequence. Analyses of the transgenic plants revealed that strong GUS activity was detected mainly in senescent leaf tissues, which is consistent with the qRT-PCR results (Figures 1C,D). These data indicated that expression of *DEAR4* is associated with natural leaf senescence.

The expression changes of *DEAR4* after exogenous phytohormones application were also studied. *DEAR4* expression was significantly up-regulated to 8 folds higher 6 h after ABA treatments and to 4 folds higher at 6 h after MeJA treatments. But no significant differences in expression of *DEAR4* were observed after SA, ACC or IAA treatment (Figure 1E).

To obtain insight into whether *DEAR4* was also involved in stress response, the expression pattern of *DEAR4* was examined in response to environmental stimuli. As shown in Figure 1F, the transcript levels of *DEAR4* increased significantly in response to NaCl, darkness and drought treatments.

### DEAR4 Is Involved in Age-Dependent Leaf Senescence

**FIGURE 2:**
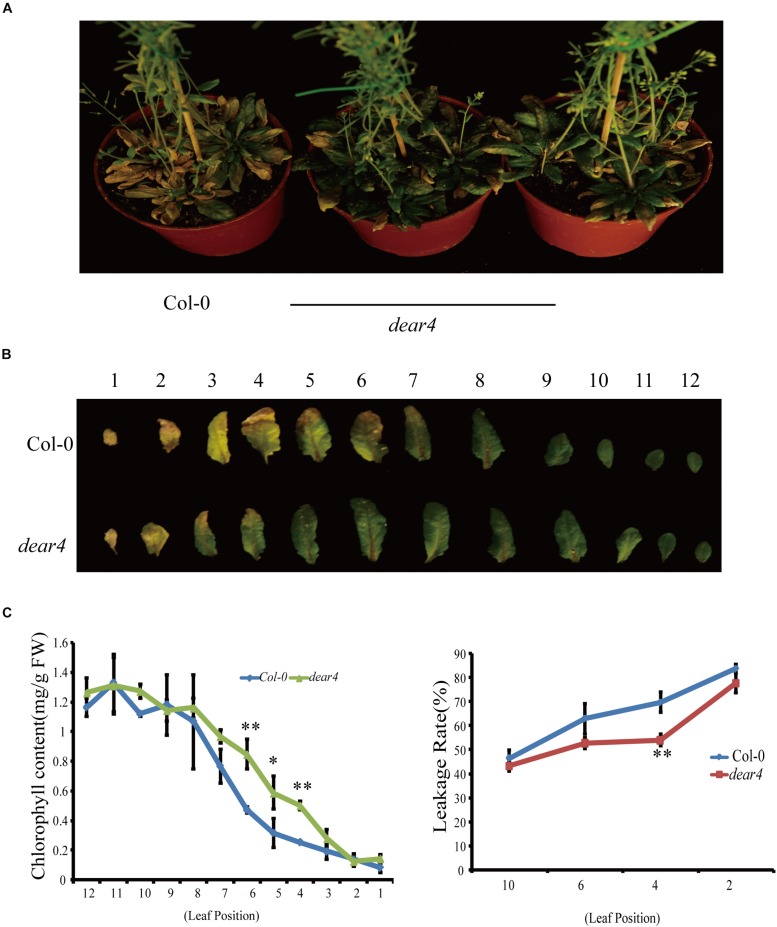
A *DEAR4* T-DNA insertion delays leaf senescence process. **(A)** Leaf phenotype of 6-week-old Col-0 and *dear4* mutant plants. **(B)** Senescence symptoms of detached leaves of Col-0 and *dear4* plants. **(C)** Total chlorophyll content (1th–12th leaf) and ion leakage rate (the leaf position as indicated) in different genotype plants as indicated. The bars are standard deviations (SD) of three biological replicates. The one and double asterisks indicate significant difference to control at the levels of 0.01 < *P* < 0.05 and *P* < 0.01 using student’s *t*-test, respectively.

To investigate the function of DEAR4, we obtained two *DEAR4* T-DNA insertion mutant lines from ABRC named *dear4* (*SALK_010653c*) and *dear4-1* (*Salk_045347*). The T-DNA insertion in the *dear4* mutant is located at the 5′UTR region of *DEAR4* (Supplementary Figure 1A) and the qRT-PCR results showed that the transcript of *DEAR4* was significantly reduced in the *dear4* mutant (Supplementary Figure 1B). Plants of *dear4* displayed a delayed senescence phenotype assessed by comparing the degree of leaf yellowing with Col-0 (Figure 2A). In 6-week-old plants, most leaves of Col-0 turned yellow with drying, yet *dear4* mutant leaves retained their integrity and displayed only partial yellowing (Figures 2A,B). Consistent with the visual phenotype, the chlorophyll content of Col-0 leaves decline faster in comparison with the counterpart of *dear4* mutant plants (Figure 2C upper). Leaf senescence often involves reduction of plasma membrane integrity, as indicated by membrane ion leakage. The delayed senescence symptoms of *dear4* can also be evidenced by lower membrane ion leakage of the leaves compared with Col-0 (Figure 2C lower). As seen in Supplementary Figure 2, *dear4-1* displayed a similar phenotype with *dear4* in delaying leaf senescence. These results demonstrated that *DEAR4* plays a potential role in promoting leaf senescence.

### Over-Expression of *DEAR4* Leads to Precocious Senescence

**FIGURE 3:**
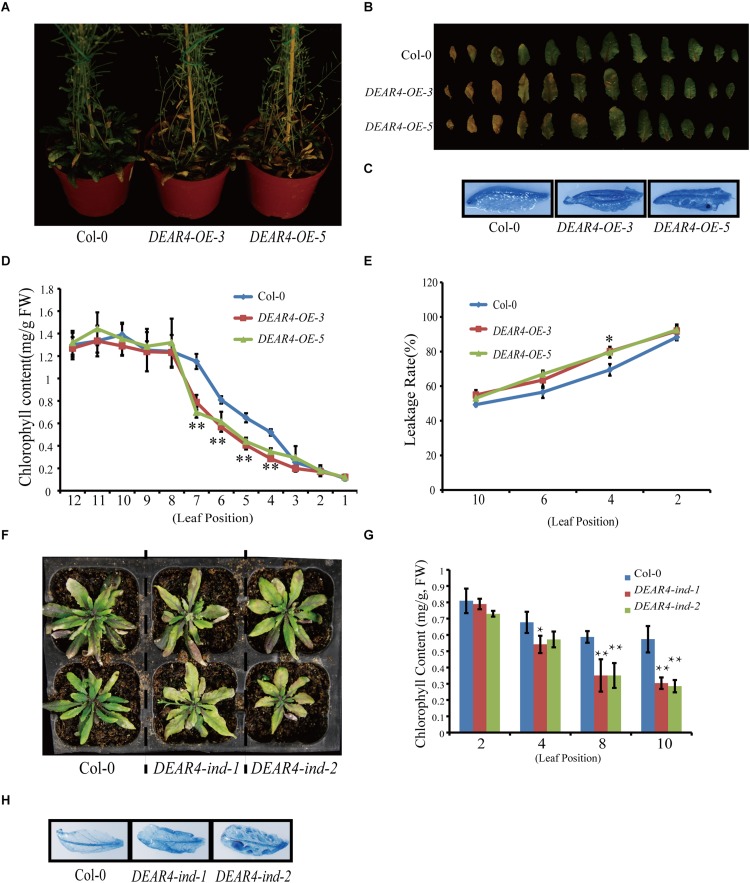
Over-expression of *DEAR4* accelerates leaf senescence. **(A)** The phenotypes of *DEAR4* over-expression lines. **(B)** Detached leaf phenotypes of Col-0, *DEAR4-OE-3*, and *DEAR4-OE-5* described in **(A)**. **(C)** Trypan blue staining of the sixth leaf of 6-week-old Col-0 and *DEAR4* over-expression lines. **(D)** Total chlorophyll content of 1th–12th leaf from 6-week-old Col-0 and *DEAR4* over-expression lines. **(E)** Leakage rate of Col-0 and *DEAR4* over-expression lines. **(F)** Inducible over-expression of *DEAR4* causes precocious senescence. **(G)** Chlorophyll content of different genotype plants that were treated with EST. **(H)** Trypan blue staining of different plants as indicated. The bars are standard deviations (SD) of three biological replicates. The one and double asterisks indicate significant difference to control at the levels of 0.01 < *P* < 0.05 and *P* < 0.01 using student’s *t*-test, respectively.

To further explore the biological function of DEAR4, we generate multiple independent *DEAR4* over-expression transgenic lines harboring the *DEAR4* CDS under control of the *CaMV 35S* promoter (*35S*::*DEAR4*). qRT-PCR analysis showed that *DEAR4* transcript levels in *DEAR4-OE-3* and *DEAR4-OE-5* were 8 to 10 folds higher than that of Col-0 (Supplementary Figure 3A). Phenotypic analysis showed that *DEAR4* over-expressing lines displayed precocious leaf senescence judged by the progression of leaf yellowing (Figures 3A,B). Consistent with the visible phenotype, the reduction in chlorophyll contents of leaves from the *DEAR4* over-expression lines were greater than in Col-0 (Figure 3D). The precocious senescence of *DEAR4* over-expression plants were supported by higher membrane ion leakage of the leaves compared with Col-0 (Figure 3E). These results demonstrated that *DEAR4* plays an important role in promoting leaf senescence. In addition, we stained the fully expanded rosette leaves of different genotype plants via Trypan blue staining to assess dead cell rates. As shown in Figure 3C, the staining of dead cells in *DEAR4* over-expression lines was higher than that in Col-0. We further examined the expression of senescence marker genes. As showed in Supplementary Figure 4, compare with Col-0, the expression levels of *SAG12*, *SEN4* were dramatically up-regulated in *DEAR4* over-expression lines but lower in *dear4* mutant, whereas the expression levels of photosynthetic gene such as *RBCS* were clearly down-regulated in *DEAR4* over-expression lines but up-regulated in the *dear4* mutant.

To further confirm the *DEAR4* gain-of-function phenotype, we investigated the phenotypes of *DEAR4* inducible over-expression lines which express *DEAR4* under the control of a β-estradiol (EST) inducible promoter (*DEAR4-ind-1* and *DEAR4-ind-2*). The expression of *DEAR4* was detected by qRT-PCR (Supplementary Figure 3B). The phenotype analysis revealed that the *DEAR4-ind* lines displayed precocious senescence after treatment with 10 μM EST compared with Col-0 (Figure 3F). A decline in chlorophyll content was observed in leaves of *DEAR4* inducible lines treated with EST (Figure 3G). Moreover, treatment with EST displayed significantly higher cell death ratio in *DEAR4* inducible gain-of-function leaves than that of control as revealed by trypan blue staining (Figure 3H). Taken together, these results indicated that DEAR4 functions in accelerating leaf senescence.

### DEAR4 Is Involved in Senescence Induced by Darkness and JA

**FIGURE 4:**
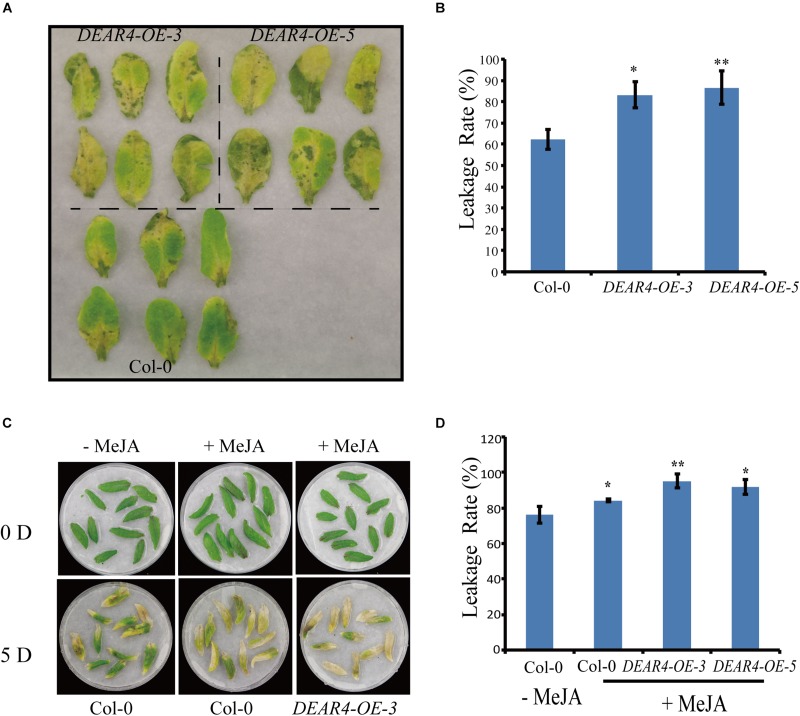
Precocious senescence phenotypes of *DEAR4* over-expression lines in dark conditions. **(A)** Phenotypes of detached leaves in response to dark condition. **(B)** Ion leakage rate in Col-0, *DEAR4-OE-3*, and *DEAR4-OE-5*. **(C,D)** *DEAR4* over-expression plants showed enhanced sensitivity to JA. The bars are standard deviations (SD) of three biological replicates. The one and double asterisks indicate significant difference to control at the levels of 0.01 < *P* < 0.05 and *P* < 0.01 using student’s *t*-test, respectively.

Given that *DEAR4* was increased at the transcriptional level under dark condition, we investigated the phenotypes of *DEAR4* over-expression plants during dark treatment. Detached leaves of Col-0 and *DEAR4* over-expression plants were covered with aluminum foil. Five days after treatment leaves from the two *DEAR4* over-expression lines exhibited an accelerated yellowing phenotype compared with those from Col-0 (Figure 4A). Consistent with the visible phenotype, *DEAR4* over-expression plants exhibited higher ion leakage rate compared with Col-0 (Figure 4B).

Since expression of *DEAR4* could be enhanced by JA treatments (Figure 1E), we sought to explore the relationship between DEAR4 and JA under dark condition. Detached leaves from 4-week-old plants of different genotypes were incubated with MeJA. After 5 days’s MeJA treatment, *DEAR4* over-expression leaves displayed serious yellowing compared with Col-0 (Figure 4C). Consistent with the visible precocious senescence, the results of ion leakage rate measurement showed that conductivity in *DEAR4* over-expression lines was higher compared with Col-0 after MeJA treatments (Figure 4D). The two independent overexpression lines displayed the similar phenotype.

### DEAR4 Functions in Response to Drought, NaCl and ABA

**FIGURE 5:**
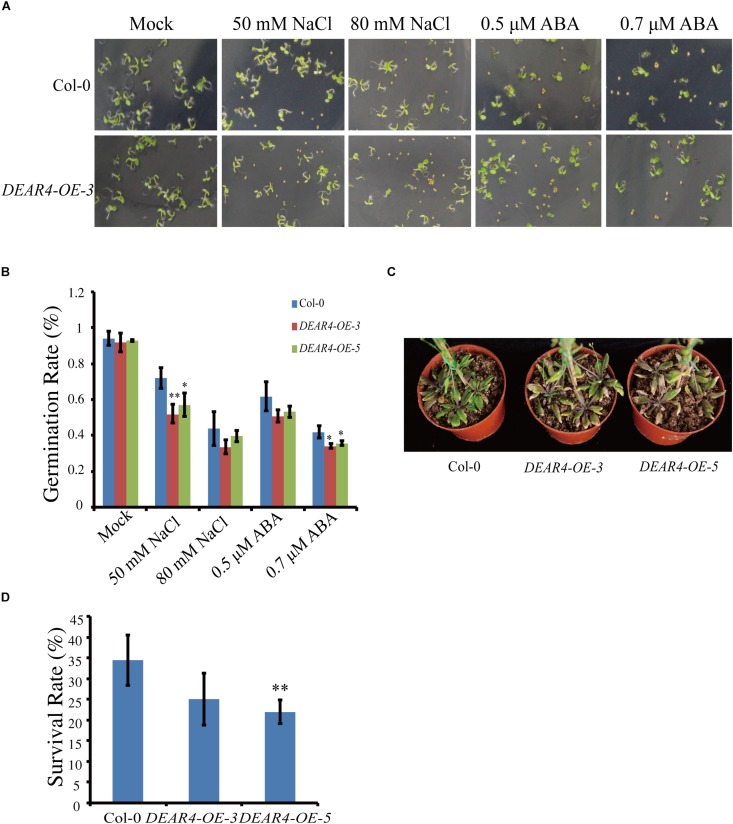
Responses of *DEAR4* over-expression plants to NaCl, ABA and drought. **(A,B)** The effect of different concentrations of NaCl and ABA on germination of Col-0 and *DEAR4* over-expression plants. **(C,D)** *DEAR4* over-expression plants were sensitive to drought treatment. The bars are standard deviations (SD) of three biological replicates. The one and double asterisks indicate significant difference to control at the levels of 0.01 < *P* < 0.05 and *P* < 0.01 using student’s *t*-test, respectively.

Based on the expression data, *DEAR4* expression can be induced by NaCl and ABA (Figures 1C,D). Seeds from *DEAR4* over-expression lines and Col-0 were sowed on 0.5× MS plates supplied with different concentrations of NaCl or ABA and the percentages of seed germination were calculated based on the number of seeds showing the radicle emergence. The results showed that the percentage of germination between the Col-0 and *DEAR4* over-expression seeds were similar under normal condition. However, in the 50 mM NaCl treatment, the radicle emerged in 71.9 and 93.2% of *DEAR4-OE-3* and Col-0 seeds, respectively. Whereas, only 40.6 and 59.9% germination rates were observed in *DEAR4* over-expression and Col-0 seeds in the 80 mM NaCl treatment. In the 0.5 μM ABA treatment, 62% of the Col-0 seeds germinated, while this rate was reduced to 53% for *DEAR4* over-expression seeds (Figures 5A,B), the two independent overexpression lines displayed the similar results. In the drought treatment, plants of the *DEAR4-OE-3*, *DEAR4-OE-5* lines and Col-0 were grown in soil for 4 weeks under normal condition. Then, drought stress was applied by withholding watering. Survival rates of plants from different genotypes were calculated after15 days of drought stress treatment. Plants of Col-0 exhibited the survival rates of over 34.37%, which was significantly higher than *DEAR4-OE-3* and *DEAR4-OE-5* transgenic plants with the survival rates of 25 and 21.87%, respectively (Figures 5C,D). Taken together, these results demonstrated that *DEAR4* conferred plant more sensitive to drought and salt stress.

### DEAR4 Regulates ROS Production

**FIGURE 6:**
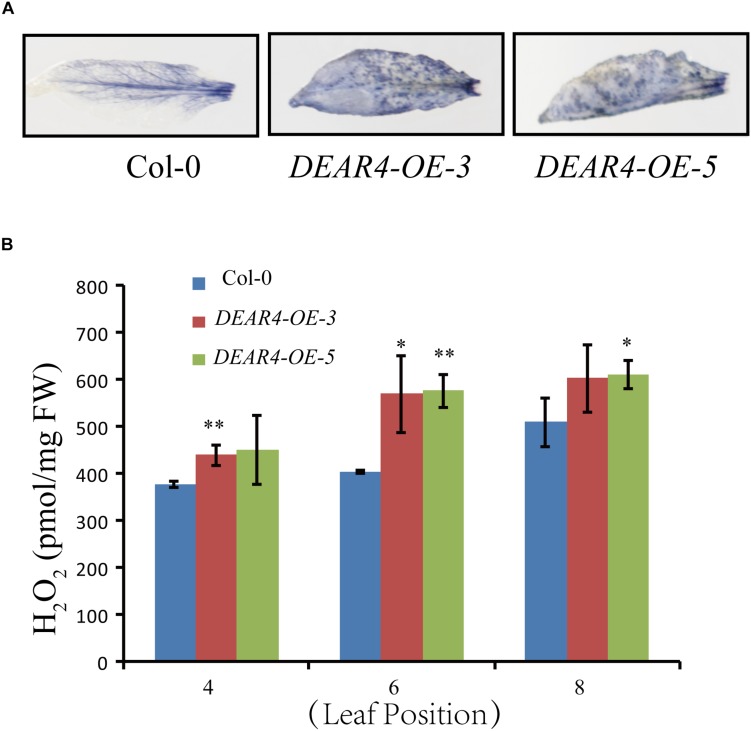
DEAR4 promotes ROS production. **(A)** NBT staining. The 5th leaves of 4-week-old plants were used in NBT staining. **(B)** Measurement of temporal accumulation of H_2_O_2_ in detached leaves of plants with different genotypes. The one and double asterisk indicate significant difference to control at the levels of 0.01 < *P* < 0.05 and *P* < 0.01 using student’s *t*-test, respectively. The bars are standard deviations (SD) of three biological replicates.

Reactive oxygen species are considered signaling molecules during leaf senescence and stress responses. To further understand the role of DEAR4 in leaf senescence and stress response, we employed NBT staining to visualize the levels of ROS. The fifth leaf from 4-week-old plants of different genotypes including *DEAR4* over-expression and Col-0 were detached and analyzed. As shown in Figure 6A, compared with Col-0, leaves of *DEAR4-OE-3* and *DEAR4-OE-5* were densely stained with dark blue and brown color by NBT staining, suggesting that DEAR4 enhanced ROS production. To further confirm the staining results, quantitative measurement was carried out to determine the endogenous H_2_O_2_ levels in plants of different genotypes. The results revealed that *DEAR4* over-expression plants accumulated significantly more H_2_O_2_ compared with Col-0 (Figure 6B).

### DEAR4 Is a Transcriptional Repressor

**FIGURE 7:**
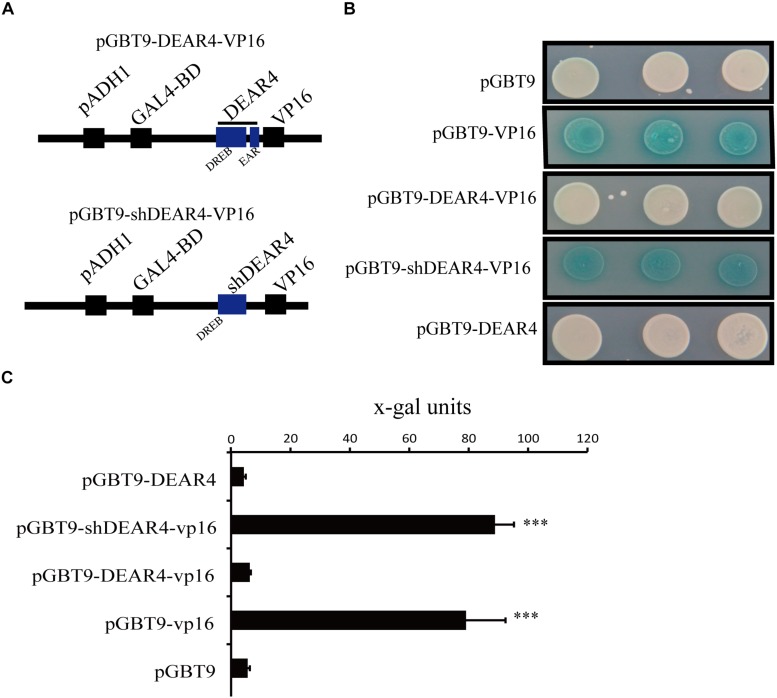
Transcriptional repression activities of DEAR4 in yeast cells. **(A)** Schematic diagram of pGBT9-DEAR4-VP16 and pGBT9-shDEAR4-VP16. **(B)** Comparison results of the Y1H assay using full length *DEAR4* or *shDEAR4* (truncated sequence absenting EAR domain coding region) fused with vp16 and control. **(C)** Liquid assay to measure x-gal activities in yeast containing different construct using CPRG as substrate. The bars are standard deviations (SD) of three biological replicates. The triple asterisk indicates significant difference to control at *P* < 0.001 using student’s *t*-test.

The Arabidopsis DEAR4 protein contains homology to the DREB1/CBF domain and the EAR motif. EAR motifs commonly played as transcriptional repression roles in plants (Yang et al., 2018). Thus, we supposed that DEAR4 has the potential of functioning as a transcriptional repressor. To verify this point, we carried out yeast one-hybrid assay. The full-length or truncated *DEAR4* (shDEAR4, without EAR domain, 1-181aa) cDNA was fused to VP16, the activation domain of a potent viral transcriptional activator. The DEAR4 (or shDEAR4)-VP16 component group was then fused downstream of the GAL4 DNA-binding domain (GAL4-BD) in the pGBT9 vector to generate the BD-DEAR4 (or shDEAR4)-VP16 construct (Figure 7A). As a positive control, yeast strain Y190 carrying the reporter genes *LacZ* transformed with pGBT9-VP16 showed strong blue reaction (Figure 7B). Similar to pGBT9-VP16, Y190 transformed with pGBT9-shDEAR4-VP16 also displayed strong blue reaction. However, Yeast strain harboring pGBT9 empty vector displayed white reaction, similar to yeast transformed with DEAR4 fused with or without VP16 (Figure 7B). These results suggest that the full length DEAR4 protein plays transcriptional repression role which is dependent on the functional EAR domain.

We also carried out liquid assay using CPRG as substrate to quantify the x-gal activities from the above described yeast one-hybrid assays. Consistent with the results from blue-white reactions, the reporter gene activity in the yeast transformed with pGBT9, BD-DEAR4, BD-DEAR4-VP16, and BD-VP16 was 5.57 ± 0.68, 4.24 ± 0.67, 6.29 ± 0.37, and 79.13 ± 13.28, respectively. However, once the EAR domain was deleted, the reporter gene activity in yeast transformed with BD-shDEAR4-VP16 was up to 88.82 ± 6.44 (Figure 7C). Taken together, BD-VP16 protein was able to induce *lacZ* expression but DEAR4 protein repressed this process. However, DEAR4 protein with EAR domain deleted lost its transcriptional repression ability.

### DEAR4 Directly Repress the Expression of *COR* and *RD29*

**FIGURE 8:**
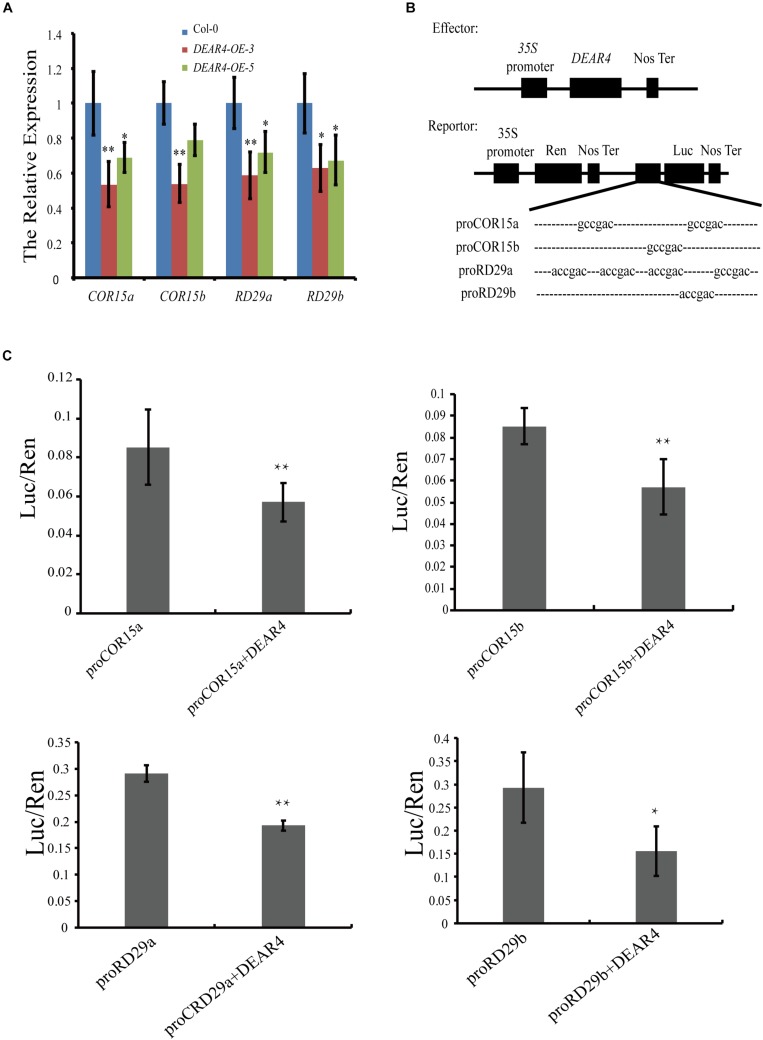
Transcriptional repression of DEAR4 on *COR* and *RD* genes. **(A)** The expression of *COR15a*, *COR15b*, *RD29a*, and *RD29b* in Col-0 and *DEAR4* over-expression lines. **(B)** Dual luciferase (LUC) constructs used in this study. Dual LUC constructs contain the double reporter and effector plasmids. In the double reporter construct, Renilla LUC expression was used to normalize the firefly luciferase activities. The effector construct in this study contains *DEAR4* driven by the *CaMV35S* promoter. **(C)** DEAR4 directly represses the expression of *COR15* and *RD29*. The *firefly LUC* gene was driven by the *COR15* and *RD29* promoters, ratio of firefly and RenLuc indicates the relative activities of promoters. The bars are standard deviations (SD) of three biological replicates. The one and double asterisk indicate significant difference to control at the levels of 0.01 < *P* < 0.05 and *P* < 0.01 using student’s *t*-test, respectively.

DEAR4 protein contains homology to the DREB1/CBF domain which binds to the DRE/CRT element containing an A/GCCGAC motif within gene promoters to regulate transcription. To further understand the molecular mechanisms underlying DEAR4’s role in leaf senescence and stress responses, we investigated whether DEAR4 could regulate some downstream genes in the DRE/CRT-mediated signaling pathway. There are two putative DRE/CRT elements (−444 to −438 bp, −267 to −261 bp) upstream of the translation start site on the *COR15a* promoter and one DRE/CRT element at −266 to −260 bp from the translation start site on the promoter of *COR15b*. Meanwhile, we identified four putative DRE/CRT elements (−353 to −347 bp, −303 to −297 bp, −246 to −240 bp, −209 to −203 bp) upstream of the translation start site on the *RD29a* promoter and one DRE/CRT element (−321 to −315 bp from the translation start site) on the promoter of *RD29b*. The expression of the *COR* and *RD* genes were significantly decreased in the *DEAR4* over-expression transgenic plants (Figure 8A), two independent overexpression lines displayed the similar results, suggesting that DEAR4 is required for reduction of the *COR* and *RD* gene expression in leaf senescence regulation.

Next, we tested whether DEAR4 directly regulates the *COR* and *RD* genes. We employed the dual luciferase strategy to investigate the direct regulation of *COR* and *RD* genes by DEAR4 in *Nicotiana benthamiana* leaves. Agrobacterium harboring the firefly luciferase-encoding gene driven by the promoter of *COR* (or *RD*) was co-transformed with or without *35S*::*DEAR4*. As an internal control, the *renilla luciferase* gene was driven by the *35S* promoter (Figure 8B). The ratio of Firefly/Renilla luciferase activities indicates the transcriptional activity of *COR* or *RD* genes. The results showed that the Firefly/Renilla luciferase ratio was significantly lower in leaves co-transformed with *DEAR4* compared to that without *DEAR4*, indicating that DEAR4 repress the expression of *COR* and *RD* genes directly (Figure 8C).

## Discussion

As the final phase of leaf development, senescence is crucial for plant survival and environmental adaptation. So far, thousands of genes and many signaling pathways have been studied for leaf senescence regulation. Among them, transcription factors are highly effective in engineering stress tolerant plants (Bengoa et al., 2019; Woo et al., 2019). A large number of transcriptional factor genes including *NAC*, *WRKY*, *MYB*, and *AP2/EREBP* were up- or down-regulated during natural and dark-induced senescence (Zhang and Zhou, 2013). Additionally, transcript profiling studies suggested that many of the genes were affected by senescence and environmental stresses at the same time (Yoshida, 2003; Sharabi-Schwager et al., 2010a; Chen et al., 2017). As the largest transcription factor family in Arabidopsis, the AP2/ERF proteins have roles in the regulation of developmental processes, hormonal signal transduction, and biotic and abiotic stress responses (Smirnoff and Bryant, 1999; Yang et al., 2019b). As one of the AP2/ERF subfamilies, members of the DREB protein family have been extensively studied over the years due to their crucial roles in regulation of abiotic- and biotic-stress responses. It’s well known that DREB protein recognized the dehydration responsive element (DRE)/C-repeat with a core sequence of A/GCCGAC to regulate gene expression (Smirnoff and Bryant, 1999; Sharoni et al., 2011; Kudo et al., 2017). More and more evidence suggested that the DREB proteins play multiple roles during plant development. The *CBF* genes including *CBF1*, −*2*, −*3*, also known as *DREB1b*, *DREB1c*, and *DREB1a*, respectively, have been identified as key regulators of cold response and drought response (Liu et al., 2004; Novillo et al., 2004). In addition, *CBF* genes also play important roles in dark induced senescence (Gilmour et al., 2000; Solanke and Sharma, 2008; Zhou et al., 2011a). Here, we investigated the function of DEAR4, which contains the DREB domain but is different from the typical DREB proteins, with an EAR motif at the C terminus. Consistent with *in silico* data in publically available databases (GENEVESTIGATOR), we found that *DEAR4* can be induced by age and dark conditions, as well as multiple abiotic stresses and hormones including ABA, JA, salt, and drought (Figure 1). More and more evidence suggested that plants have the capacity of integrating various signaling pathways to provide a greater regulatory potential for enhancing environment adaption. Our data revealed that DEAR4 was involved in age and dark induced leaf senescence based on phenotype and physiological data (Figures 2–4). At the molecular level, the precocious leaf senescence phenotype is associated with a gradual increase in the transcript levels of *SAG*s including *SAG12*, *SEN4* and decrease in the expression of the *RBCS* gene (Supplementary Figure 2). Previous study has reported that DEAR1, one of the DEAR4 homologs, plays roles in mediating crosstalk between biotic and abiotic stresses. *DEAR1* expression was enhanced by pathogen infection and cold treatment. Over-expression of *DEAR1* caused an activated defense phenotype. Additionally, the induction of DREB1/CBF family genes by cold treatment was suppressed in *DEAR1* over-expression lines, leading to a reduction in freezing tolerance (Tsutsui et al., 2009). In addition, DEAR1 is included in the A-5 subgroup of the DREB/family that could form a negative feedback regulation of the DREB1/CBF and DREB2 pathway in response to cold and dehydration (Mizoi et al., 2012). There are five DEAR1 homologs including DEAR4 within the Arabidopsis genome that also contain a DREB domain and an EAR motif. The role of DEAR4 in stress response therefore is not surprising. Interestingly, we found that DEAR4 also plays a role in leaf senescence, suggesting that the DEAR genes may play different roles in plant development and response to environmental stimuli. It will be interesting to find out whether the similar negative feedback mechanism exists in DEAR4’s function in leaf senescence and stress response.

Jasmonic acid acts as a crucial signal to modulate multiple plant processes including senescence and stress responses. JA content was much higher in senescent leaves than in non-senescent ones (Shan et al., 2011; Seltmann and Berger, 2013). Additionally, exogenous application of JA enhances plants’ freezing tolerance with or without cold acclimation. Further study revealed that JA positively regulates *CBF* to up-regulate downstream cold-responsive genes to enhance cold tolerance (Hu et al., 2013, 2017). JAZ proteins were discovered as repressors of JA signaling through the COI1-dependent 26S proteasome pathway for protein degradation. Once JAZ proteins were reduced, various downstream transcription factors including MYC2, MYC3 and MYC4 were activated (Fernandez-Calvo et al., 2011; Hu et al., 2013). In darkness, the mutant of *JAZ7* partially liberated *MYC2*/*MYC3*/*MYC4* from suppression, resulting in the up-regulation of the downstream genes related to indole-glucosinolate biosynthesis, sulfate metabolism, callose deposition, and JA-mediated signaling pathways (Yu et al., 2016). In this study, we found that *DEAR4* was induced by exogenous MeJA (Figure 1E), meanwhile, DEAR4 can enhance the role of MeJA in promoting senescence (Figures 4C,D), which provide clues that *DEAR4* may play roles in JA regulating senescence and stress responses. The DEAR4 protein contains the EAR motif which plays an important role in ethylene-responsive transcriptional regulation. Interestingly, previous studies have reported the mechanism of crosstalk between JA and other plant hormones including ethylene in plant growth and stress responses. JA and ethylene antagonize or coordinately regulate plant stress response (Zhu, 2014; Yang et al., 2019a). JA and ethylene pathways most likely crosstalk at the levels of JAZ–EIN3 and JAZ–EIL1 (Zhu et al., 2011; Kazan and Manners, 2012; Zhang et al., 2014). The EAR domain proteins could be involved in JA signal pathway. For example, the NINJA (Novel Interactor of JAZ, an EAR motif containing protein) mediated JAZ pathway to block the activity of MYC2, repressing the JA-dependent root growth inhibition and defense processes (Li et al., 2019). Both JA and ethylene were involved in leaf senescence. So far, the crosstalk between JA and ethylene’s functions in leaf senescence is not clear. Based on our data, DEAR4 could potentially play a role in both JA and ethylene pathways in regulating leaf senescence.

Plant ROS include hydrogen peroxide (H_2_O_2_), superoxide anion (O^2–^), hydroxyl radicals (OH) and singlet oxygen (1O_2_), which can be produced from chloroplast and mitochondrial electron transport chains, and oxidases and peroxidases located in the peroxisomes or in the plasmalemma/apoplast (Wang et al., 2013). ROS are not only as by-products of metabolic pathways but also play signaling roles during normal plant development. Multiple stresses are known to enhance ROS generation (Apel and Hirt, 2004; Mittler et al., 2004). The senescence process also increases accumulation of ROS (Jajic et al., 2015; Li et al., 2016). Thus, ROS production regulation plays key roles in both senescence and stress responses. Given that DEAR4 was involved in age and dark induced senescence, we hypothesized that DEAR4 may be involved in regulating leaf senescence though the proliferation of ROS. Consistent with this hypothesis, over-expression of *DEAR4* can induce the production of ROS. *DEAR4* over-expression lines displayed substantially induced ROS production based on the NBT staining and H_2_O_2_ content measurement data (Figure 6). Leaf senescence is commonly associated with electrolyte leakage which represents loss of membrane integrity. Excessive production of ROS results in membrane lipid peroxidation. Our results demonstrated that DEAR4 was involved in age- and dark induced leaf senescence and multiple stresses responses all of which are associated with membrane damage and accumulation of ROS, suggesting a crucial role of DEAR4 in these processes potentially via ROS regulation.

Leaf senescence is also regulated by environmental stimuli including salinity, drought, low quality light and darkness (Buchanan-Wollaston et al., 2005). Numerous *SAG*s were influenced by diverse abiotic and biotic stresses (Quirino et al., 1999; Weaver and Amasino, 2001). Additionally, some of the genes that regulate stress responses may also have an important role in regulating leaf senescence (Binyamin et al., 2001). For example, among the 43 transcription factor genes up-regulated during senescence, 28 were also induced by various stresses (Lim et al., 2007). The DREB protein family is known to regulate abiotic stress responses in plants. The DEAR4 protein contains a DREB domain. The expression of *DEAR4* is lower in young leaves, but is up-regulated in senescing leaves. Meanwhile, *DEAR4* gene transcript was induced by senescence as well as senescence-stress associated hormones including ABA and JA (Figure 1). Detached leaves of *DEAR4* gain-of-function plants accelerated the senescence process induced by age, darkness or MeJA, suggesting that DEAR4 possibly integrates the age-dependent leaf senescence with responses to environmental stimuli including darkness and phytohormones. A large amount of evidence suggested that the *DREB* genes play a major role in cold- and osmotic-stress signal transduction pathways by recognizing the dehydration responsive element (DRE)/C-repeat with a core sequence A/GCCGAC. In this study, we observed that DEAR4 exhibited transcriptional repression activities in yeast cells depending on its EAR motif (Figure 7). As expected, DEAR4 was demonstrated to be able to directly repress the expression of DRE element genes *COR* and *RD* genes (Figure 8). Interestingly, it has been reported that COR and RD genes were involved in senescence and stress responses (Lee and Seo, 2015; Bremer et al., 2017; Shi et al., 2017b).

## Data Availability Statement

All datasets generated for this study are included in the article/Supplementary Material.

## Author Contributions

YG conceived the project. ZZ and YG designed the research, performed the data analysis, and wrote the manuscript. ZZ, WL, MX, and XG performed the experiments.

## Conflict of Interest

The authors declare that the research was conducted in the absence of any commercial or financial relationships that could be construed as a potential conflict of interest.

## Footnotes

1 https://www.genevestigator.com/gv/index.jsp
