# Supplementary material for: DEAR4, a Member of DREB/CBF Family, Positively Regulates Leaf Senescence and Response to Multiple Stressors in Arabidopsis thaliana
Source: Front Plant Sci. 2020 Mar 31;11:367. doi: 10.3389/fpls.2020.00367 (PMC7136848; doi:10.3389/fpls.2020.00367)
Supplement: Supplementary file 1 [file Data_Sheet_1.docx]

Supplementary Material

**SUPPLEMENTARY FIGURE LEGENDS**

**Supplementary Figure 1. Genotyping of the *dear4* mutant.** (A) Gene structure and *dear4* (*SALK_010653C*)T-DNA insertion in the 5’UTR region. Exon is shown as black box. (B)Transcript levels of *DEAR4* were examined by qRT-PCR using RNA samples extracted from 4-week-old plants. The bars are standard deviations (SD) of three biological replicates.

**Supplementary Figure 2. Phenotypes of *dear4-1*.** (A) Transcript levels of *DEAR4* were examined by qRT-PCR using RNA samples extracted from 4-week-old plants. The bars are standard deviations (SD) of three biological replicates. (B) The phenotype of *dear4-1* compared with Col-0. (C) Chlorophyll content was determined from the sixth leaf.

**Supplementary Figure 3. The expression of *DEAR4* in plants with different genotypes**. (A) The expression of *DEAR4* in *col*, *DEAR4-OE-3* and *DEAR4-OE-5.* (B) The expression of *DEAR4* in *col*, *DEAR4-ind-1* and *DEAR4-ind-2*. Plants of *col, DEAR4-ind-1* and *DEAR4-ind-2* grown in continuously light condition were sprayed with 20 μM EST and incubated for additional 12 hours before RNA extraction. The bars are standard deviations (SD) of three biological replicates.

**Supplementary Figure 4. The expression of *SEN4*, *SAG12* and *RBCS* in plants with different genotypes**. mRNA level was determined by qPCR using total RNA. The *actin2* was used as the internal control. The bars are standard deviations (SD) of three biological replicates.
