# Supplementary figures and images for: DEAR4, a Member of DREB/CBF Family, Positively Regulates Leaf Senescence and Response to Multiple Stressors in Arabidopsis thaliana
Source: Front Plant Sci. 2020 Mar 31;11:367. doi: 10.3389/fpls.2020.00367 (PMC7136848; doi:10.3389/fpls.2020.00367)

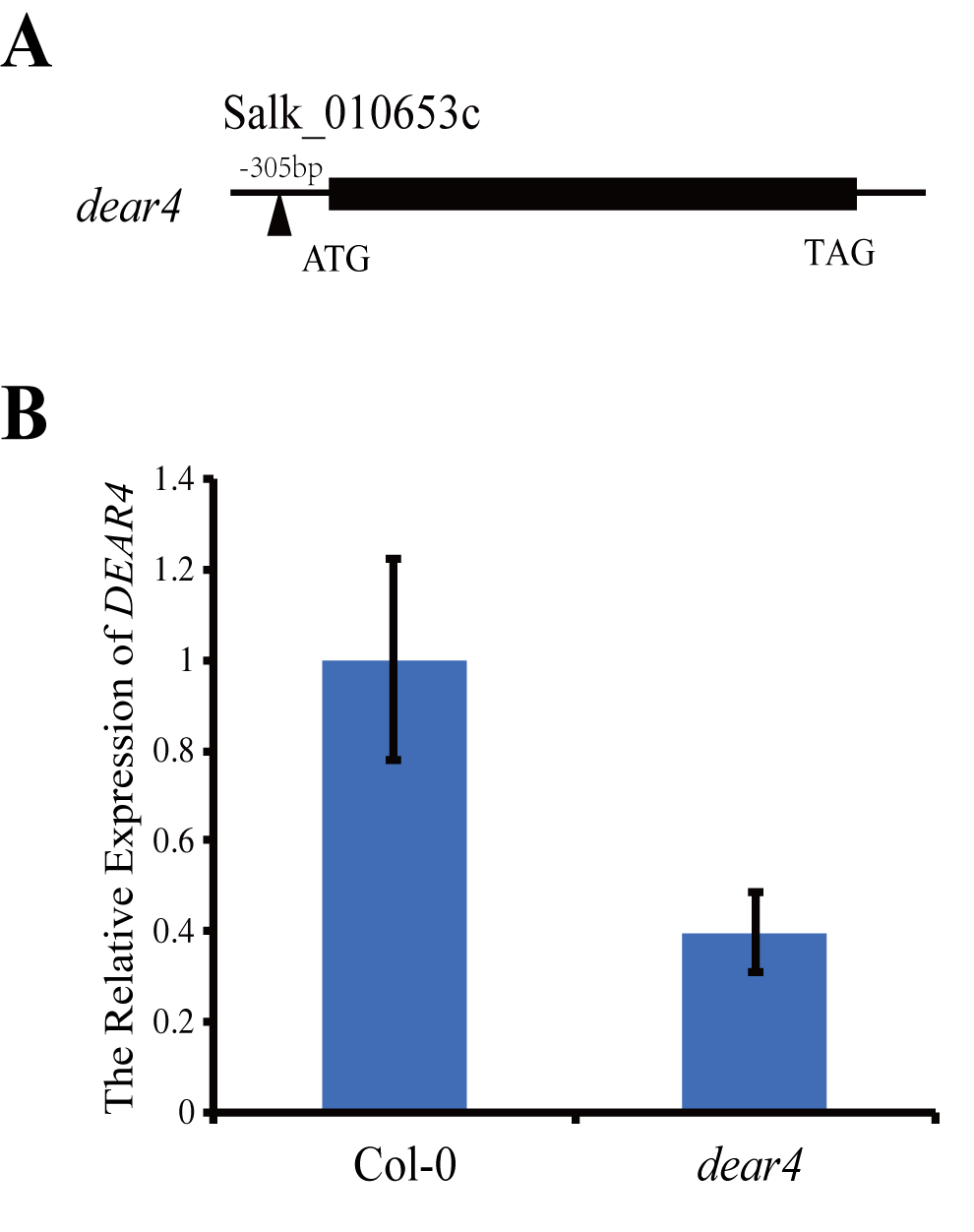

Supplement: Supplementary file 2 [file Image_1.TIF]

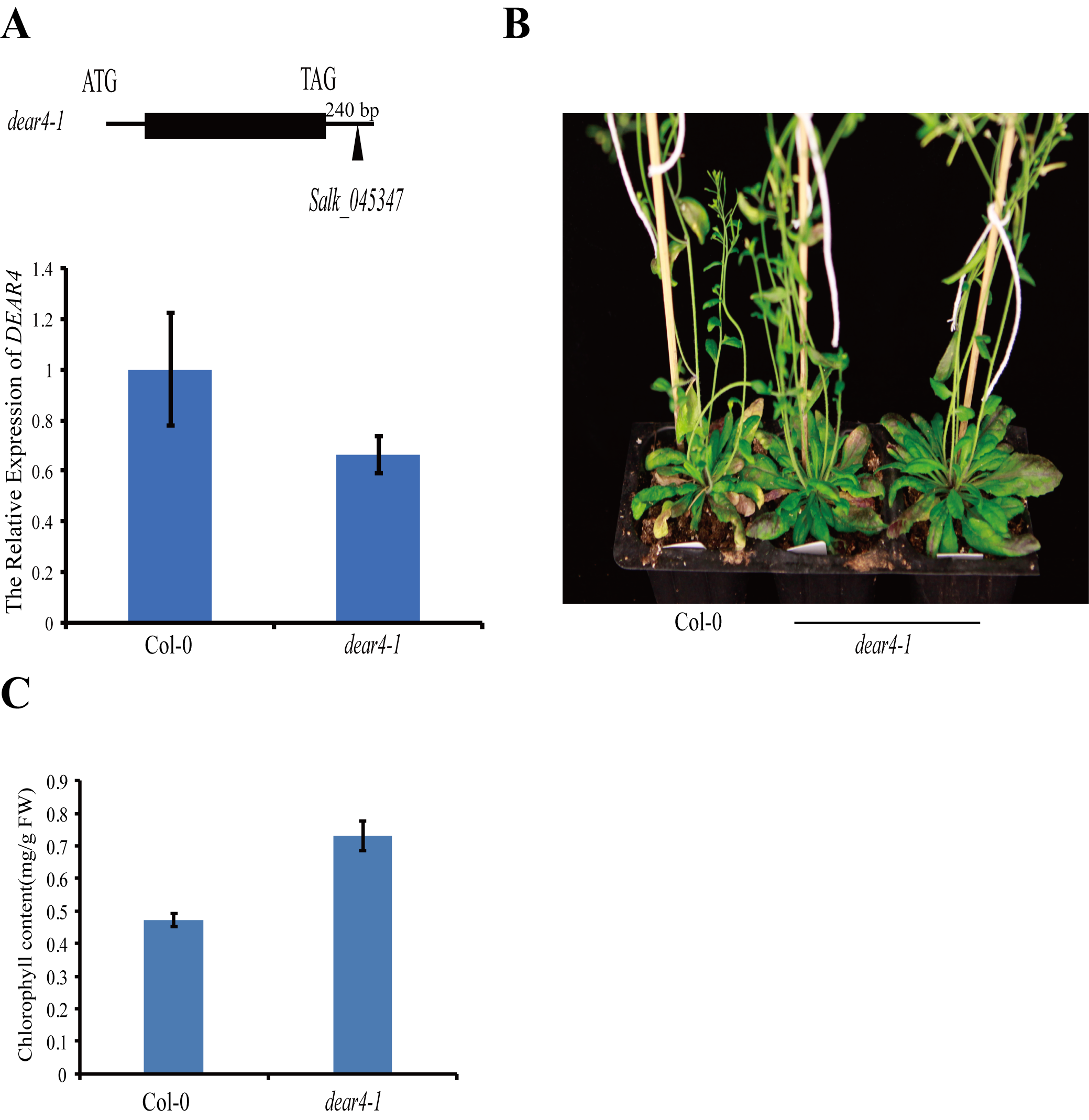

Supplement: Supplementary file 3 [file Image_2.TIF]

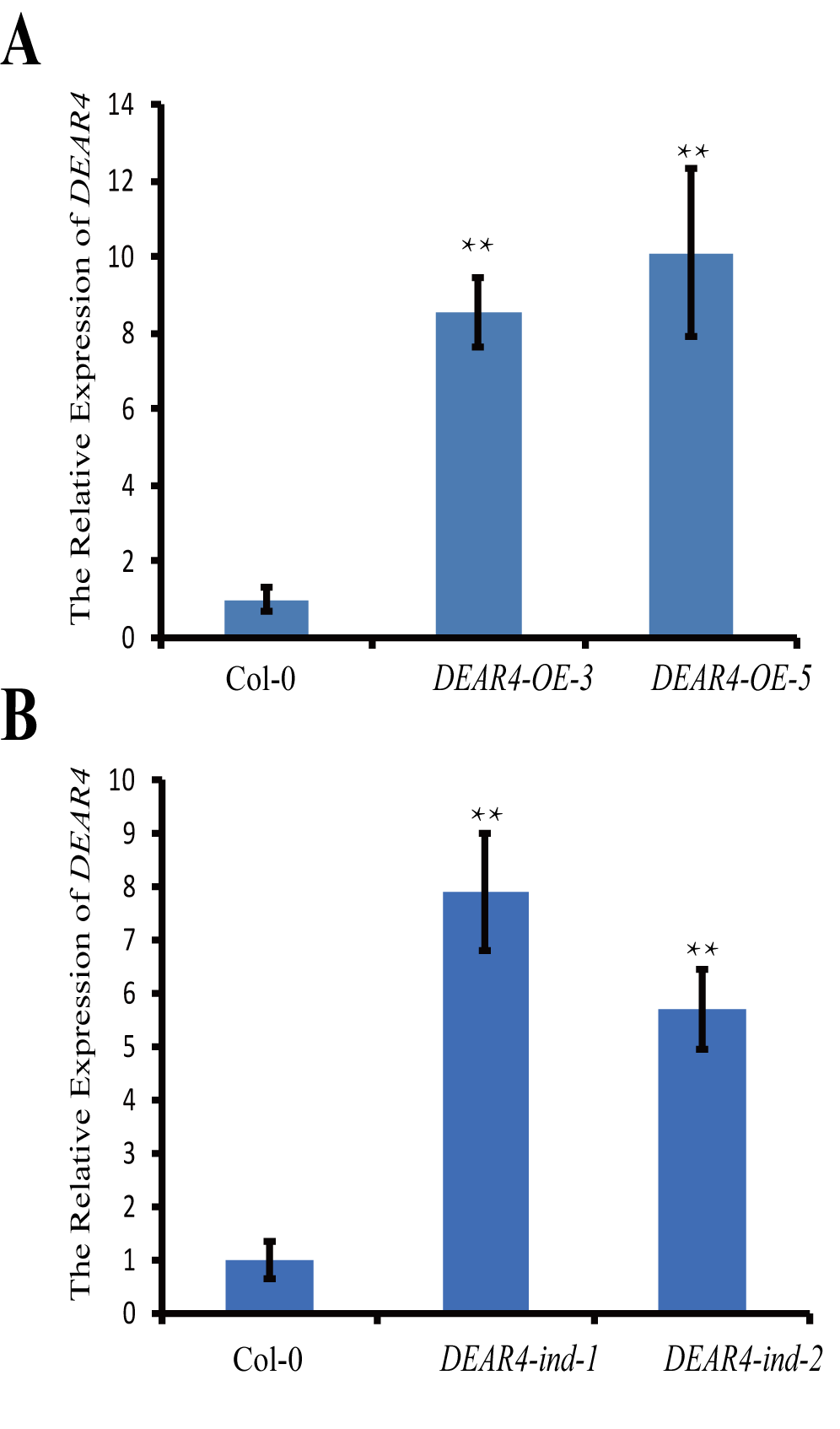

Supplement: Supplementary file 4 [file Image_3.TIF]

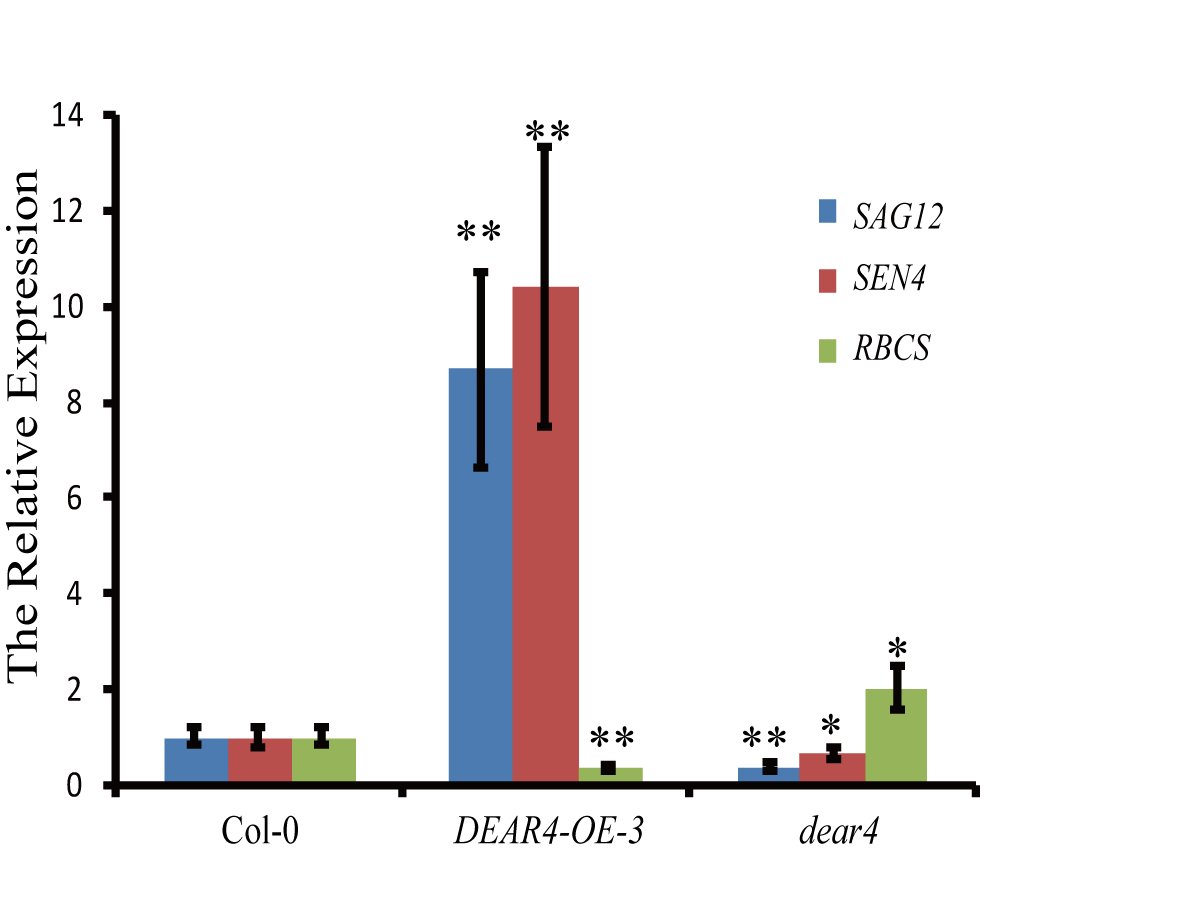

Supplement: Supplementary file 5 [file Image_4.TIF]
